# Non‐Additive Effects of Combined NOX1/4 Inhibition and Calcimimetic Treatment on a Rat Model of Chronic Kidney Disease‐Mineral and Bone Disorder (CKD‐MBD)

**DOI:** 10.1002/jbm4.10600

**Published:** 2022-02-11

**Authors:** John G Damrath, Neal X Chen, Corinne E Metzger, Shruthi Srinivasan, Kalisha O'Neill, Annabel Biruete, Keith G Avin, Joseph M Wallace, Matthew R Allen, Sharon M Moe

**Affiliations:** ^1^ Weldon School of Biomedical Engineering Purdue University West Lafayette IN USA; ^2^ Division of Nephrology, Department of Medicine Indiana University School of Medicine Indianapolis IN USA; ^3^ Department of Anatomy and Cell Biology Indiana University School of Medicine Indianapolis IN USA; ^4^ Department of Physical Therapy Indiana University School of Health and Rehabilitation Sciences Indianapolis IN USA; ^5^ Department of Biomedical Engineering Indiana University‐Purdue University at Indianapolis Indianapolis IN USA; ^6^ Department of Medicine Roudebush Veterans Administration Medical Center Indianapolis IN USA

**Keywords:** BONE MICROARCHITECTURE, CALCIMIMETICS, CHRONIC KIDNEY DISEASE‐MINERAL AND BONE DISORDER, OXIDATIVE STRESS, VASCULAR CALCIFICATION

## Abstract

Chronic kidney disease‐mineral and bone disorder (CKD‐MBD) increases cardiovascular calcification and skeletal fragility in part by increasing systemic oxidative stress and disrupting mineral homeostasis through secondary hyperparathyroidism. We hypothesized that treatments to reduce reactive oxygen species formation and reduce parathyroid hormone (PTH) levels would have additive beneficial effects to prevent cardiovascular calcification and deleterious bone architecture and mechanics before end‐stage kidney disease. To test this hypothesis, we treated a naturally progressive model of CKD‐MBD, the Cy/+ rat, beginning early in CKD with the NADPH oxidase (NOX1/4) inhibitor GKT‐137831 (GKT), the preclinical analogue of the calcimimetic etelcalcetide, KP‐2326 (KP), and their combination. The results demonstrated that CKD animals had elevated blood urea nitrogen, PTH, fibroblast growth factor 23 (FGF23), and phosphorus. Treatment with KP reduced PTH levels compared with CKD animals, whereas GKT treatment increased C‐terminal FGF23 levels without altering intact FGF23. GKT treatment alone reduced aortic calcification and NOX4 expression but did not alter the oxidative stress marker 8‐OHdG in the serum or aorta. KP treatment reduced aortic 8‐OHdG and inhibited the ability for GKT to reduce aortic calcification. Treatments did not alter heart calcification or left ventricular mass. In the skeleton, CKD animals had reduced trabecular bone volume fraction and trabecular number with increased trabecular spacing that were not improved with either treatment. The cortical bone was not altered by CKD or by treatments at this early stage of CKD. These results suggest that GKT reduces aortic calcification while KP reduces aortic oxidative stress and reduces PTH, but the combination was not additive. © 2022 The Authors. *JBMR Plus* published by Wiley Periodicals LLC on behalf of American Society for Bone and Mineral Research.

## Introduction

Chronic kidney disease (CKD) affects 15% of Americans and results in numerous biochemical changes including elevated fibroblast growth factor 23 (FGF23) and parathyroid hormone (PTH).^(^ ^1^, ^2^ ^)^ Although these hormonal alterations are initially effective at maintaining mineral homeostasis, sustained elevations in FGF23 and PTH are thought to mediate the pathological sequalae that eventually result in CKD‐mineral and bone disorder (CKD‐MBD).^(^ ^3^, ^4^ ^)^ Specifically, patients with CKD‐MBD demonstrate arterial and soft tissue calcification and altered bone microarchitecture with reduced mechanical integrity.^(^ ^5^, ^6^, ^7^ ^)^ Clinically, these changes manifest as increased cardiovascular mortality, fractures, and fracture‐related mortality across all stages of CKD compared with the age‐ and sex‐matched population with normal kidney function.^(^ ^8^ ^)^ Treatments to reduce the risk of cardiovascular‐ and fracture‐related mortalities in CKD are needed.

To date, calcium‐based phosphate binders, activated vitamin D, and calcimimetics have shown efficacy in lowering PTH levels in CKD patients.^(^ ^9^, ^10^ ^)^ However, vascular calcification is more prevalent in patients receiving calcium‐based phosphate binders, and no studies have demonstrated improvements in fracture risk in patients receiving activated vitamin D.^(^ ^11^, ^12^ ^)^ In contrast, a secondary analysis of the Evaluation of Cinacalcet HCl Therapy to Lower Cardiovascular Events (EVOLVE) trial has demonstrated that calcimimetics may reduce fractures in CKD patients.^(^ ^13^ ^)^ Studies in animals have also demonstrated that calcimimetics can prevent the progression of medial and atherosclerotic calcification,^(^ ^14^, ^15^ ^)^ and studies in humans show a trend toward reduction in coronary artery calcification.^(^ ^16^ ^)^ These agents act by sensitizing the calcium‐sensing receptor on chief cells of the parathyroid gland, resulting in decreased PTH synthesis and secretion and similarly affect calcium signaling in multiple other organs, including vascular smooth muscle cells (VSMC) (reviewed in Diaz‐Soto and colleagues^(17^ ^)^). Therefore, calcimimetics may represent a promising treatment strategy for reducing fractures and vascular calcification in CKD patients with secondary hyperparathyroidism but require further investigation.

CKD patients also have increased oxidative stress that may compound or accelerate the end organ response to the biochemical changes of CKD‐MBD.^(^ ^18^ ^)^ In CKD, dialysis and uremic toxins, such as indoxyl sulfate, can activate inflammatory cells to release nuclear factor kappa‐light‐chain‐enhancer of activated B cells (NF‐κB) and tumor necrosis factor alpha (TNFα).^(^ ^19^, ^20^ ^)^ Downstream, these metabolites increase nicotinamide adenine dinucleotide phosphate (NADPH) oxidase (NOX) activity in VSMCs, producing superoxide radicals via the transfer of electrons from NADPH to oxygen.^(^ ^20^ ^)^ NOX4 activity in VSMCs has been found to increase during vascular calcification, induce osteochondrogenic transformation of VSMCs, and is linked to increased patient mortality in CKD.^(^ ^21^, ^22^, ^23^ ^)^ Therefore, reactive oxygen species produced by NOX4 may be important drivers of the vascular calcification found in CKD‐MBD.

The goal of this study was to evaluate the effects of the NOX1/4 inhibitor GKT‐137831 (GKT), the PTH‐lowering calcimimetic KP‐2326, and their combination in a slowly progressive rat model of CKD, the male Cy/+ rat, at levels of moderate CKD to prevent end organ manifestations of CKD‐MBD. We hypothesized that lowering PTH (KP‐2326) and reducing oxidative stress (GKT) would each have individual, and the combination would have additive, beneficial effects on bone and vascular health in CKD.

## Materials and Methods

### Experimental design

Cy/+ rats are characterized by an autosomal dominant progressive cystic kidney disease. The animals have a mutation in *Anks6*, a gene that codes for the protein SamCystin located at the base of cilia, but not in the cilia. In male Cy/+ rats, CKD‐MBD develops spontaneously and progresses to end‐stage kidney disease by 30 to 40 weeks of age. Female rats, however, do not develop azotemia, even as old as 80 weeks^(^ ^24^ ^)^ or after oophorectomy.^(^ ^25^ ^)^ Therefore, only male rats were used in the present study. The Cy/+_IU_ colony of rats has been bred at Indiana University for over 20 years. For the present study, male Cy/+_IU_ rats (hereafter called CKD) and unaffected Sprague Dawley normal littermates were placed on a casein diet (Envigo Teklad, Indianapolis, IN, USA; TD.04539; 0.7% Pi, 0.6% Ca) at 18 weeks of age to produce a more consistent CKD‐MBD phenotype.^(^ ^26^ ^)^ Five groups of animals were compared (*n* = 14 per group): (i) normal littermate animals; (ii) CKD animals injected with vehicle (DMSO); (iii) CKD animals treated with GKT‐137831 in DMSO (60 mg/kg, s.c. injection daily); (iv) CKD animals treated with the calcimimetic KP‐2326 in PBS, a preclinical form of etelcalcetide (0.6 mg/kg, i.p. injection thrice/week); and (v) CKD animals treated with GKT‐137831 plus KP‐2326.

The dose of GKT was determined from published studies.^(^ ^27^, ^28^ ^)^ The dose of KP‐2326 was determined by preliminary dose finding studies to determine optimal dose for maximizing PTH suppression without profound drop in serum calcium. Treatment began at 18 weeks of age (~60% normal glomerular filtration rate [GFR]) for 10 weeks. At 28 weeks of age (~30% normal GFR), animals were anesthetized with isoflurane and underwent cardiac puncture for blood collection followed by exsanguination and bilateral pneumothorax to ensure death. The heart, aorta, kidney, tibias, and femora were collected from each animal, weighed as appropriate, and stored for analysis. The femora were kept frozen at −20°C in PBS‐soaked gauze until use. Left ventricular mass index (LVMI) was determined by dividing total heart weight by body weight. All procedures were approved by the Indiana University School of Medicine's Institutional Animal Care and Use Committee before initiating the study.

### Blood biochemistry

Blood plasma/serum was analyzed for blood urea nitrogen, creatinine, calcium, and phosphorus using colorimetric assays (Pointe Scientific, Canton, MI, USA, or BioAssay Systems, Hayward, CA, USA). Plasma intact PTH, serum C‐terminal, and intact FGF23 were determined by ELISA kits (Quidel, San Diego, CA, USA). Serum levels of the oxidative stress marker 8‐hydroxy‐2′‐deoxyguanosine (8‐OHdG) were measured using an ELISA kit (Enzo Life Sciences, Farmingdale, NY, USA).^(^ ^29^ ^)^

### Aortic arch and heart calcification and oxidative stress

To quantify aortic arch and heart calcification, segments of the aortic arch and heart were incubated in 0.6 N HCl for 72 hours. The supernatant was analyzed for calcium using the *o*‐cresolphthalein complex 1 method (calcium kit; Pointe Scientific) and normalized by tissue dry weight as previously described.^(^ ^22^, ^23^, ^30^, ^31^ ^)^ Tissue oxidative stress of the aortic arch and heart was assessed by 8‐OHdG using an ELISA kit (Enzo Life Sciences).

### RNA isolation, quantification, and real‐time PCR

Total RNA from the aorta was isolated using the miRNeasy Mini Kit (Qiagen, Valencia, CA, USA). Target‐specific PCR primers were obtained from Applied Biosystems (Carlsbad, CA, USA). The gene expression of NADPH oxidase isoforms 2 and 4 (NOX2 and 4) were analyzed to assess isoform specificity by real‐time PCR with Taqman gene expression assays (TaqMan MGP probes, FAM dye‐labeled, Applied Biosystems) using ViiA 7 systems. The cycle number at which the amplification plot crosses the threshold was calculated (C_T_), and the ∆∆C_T_ method was used to analyze the relative changes in mRNA expression and normalized by beta‐actin as previously described.^(^ ^22^, ^31^ ^)^

### Bone assessments

Micro‐computed tomography (micro‐CT) was performed using micro‐CT (Skyscan 1172; Bruker microCT, Kontich, Belgium) at 12‐micron resolution using methods previously published.^(^ ^31^ ^)^ Briefly, a 1 mm region of interest of the proximal tibia starting approximately 0.5 mm from the distal end of the growth plate was used for analysis. Cortical bone parameters were assessed from 5 slices taken at 4 mm distal to the end of the trabecular region. Trabecular bone volume fraction (BV/TV, %) and cortical porosity were quantified using CT analyzer (CTan). Whole femora were scanned (Skyscan 1176) at 18‐micron resolution to assess a subset of geometric properties at the mid‐diaphysis for mechanical analysis using a custom MATLAB script. These properties include the distance from the centroid to the anterior bone surface (c) and the bending moment of inertia about the medial‐lateral axis (I_ML_). All CT analyses were done in accordance with standard guidelines.^(^ ^32^ ^)^

Femoral mechanical properties were assessed in 4‐point bending on a mechanical testing system (TestResources, Shakopee, MN, USA). Bones were thawed to room temperature, hydrated in saline, and then placed anterior surface down on bottom supports (span = 18 mm). The upper supports (span = 7 mm) were brought down in contact with the specimen's posterior surface, and then testing was conducted at a displacement rate of 0.03 mm/s. Force versus displacement data were collected at 10 Hz, and structural parameters were determined from curves using a custom MATLAB script. Material properties were estimated using the values of c and I_ML_ taken from the fracture site and standard beam‐bending equations.^(^ ^33^ ^)^

### Statistics

Statistical analyses were conducted by first excluding outliers using ROUT (Q = 1%), followed by a normality test (*p* < 0.05 with Anderson‐Darling test). If normality testing was fulfilled, we used a one‐way ANOVA and, if overall ANOVA showed *p* < 0.05, we conducted within‐group comparisons by Dunnett's post hoc analyses, comparing untreated CKD rats versus each of the other groups. If normality was not fulfilled, then the data for each group was first log transformed, normality confirmed, and then the identical analyses as above performed. The results are expressed as means ± standard deviation (SD) with *p* < 0.05 considered significant (GraphPad Prism Software, La Jolla, CA, USA).

## Results

### The effects of GKT and KP on CKD‐MBD biochemistries

**Fig. 1:**
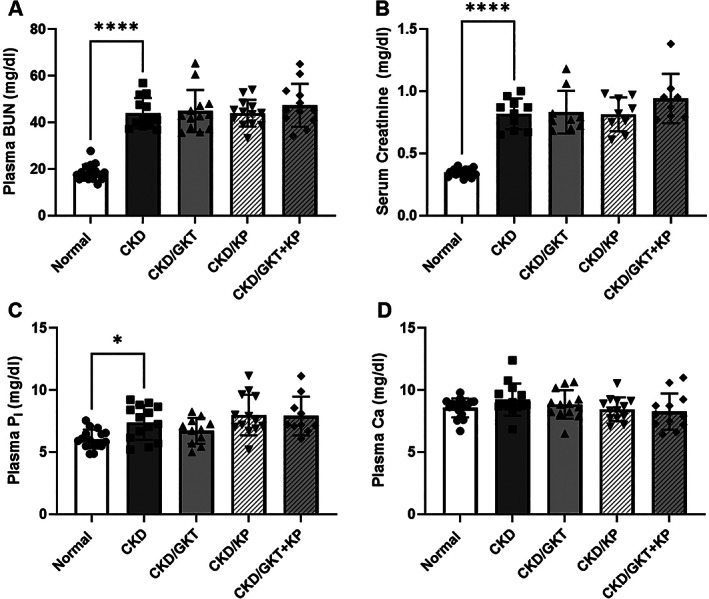
Plasma**/**serum markers of chronic kidney disease (CKD) in 28‐week‐old Cy/+ rats. BUN (*A*), creatinine (*B*), and phosphorus (*C*) were elevated in CKD animals compared with normal. Calcium levels were not altered in CKD animals (*D*). No biochemistries were altered by treatment. Data are shown as mean ± SD and analyzed by one‐way ANOVA. If *p* < 0.05, Dunnett's multiple comparison test was performed for each group versus CKD: *p < 0.05, ****p < 0.0001. BUN = blood urea nitrogen; P_i_ = inorganic phosphate; Ca = calcium.

**Fig. 2:**
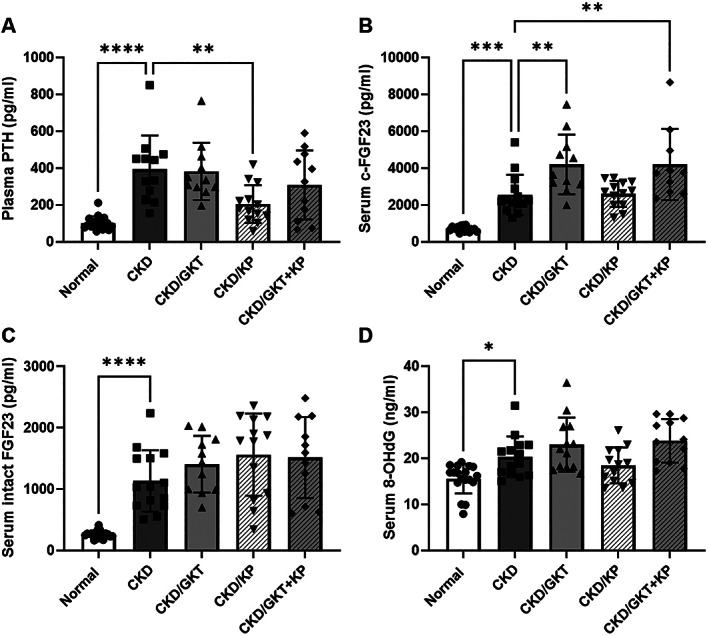
Plasma**/**serum markers of mineral homeostasis and oxidative stress in 28‐week‐old Cy/+ rats. PTH was significantly elevated in CKD animals and lowered by KP treatment alone (*A*). cFGF23 levels were elevated in CKD and further elevated by GKT treatment (*B*), whereas intact levels were increased in CKD but not altered by the treatments (*C*). The oxidative stress marker 8‐OHdG was elevated in the serum of CKD animals (*D*). Data are shown as mean ± SD and analyzed by one‐way ANOVA. If *p* < 0.05, Dunnett's multiple comparison test was performed for each group versus CKD: *p < 0.05, **p < 0.01, ***p < 0.001, ****p < 0.0001. PTH = parathyroid hormone; FGF23 = fibroblast growth factor 23; 8‐OHdG = 8‐hydroxy‐2′‐deoxyguanosine.

The present study utilized a slowly progressive model of CKD, the Cy/+ rat, to determine the effects of treatments initiated in mild CKD (approximately stage 3 CKD, glomerular filtration rate approximately 60% of normal) with follow‐up to CKD stage 4. There were no differences in body weight between the animal groups. There was impaired kidney function at the endpoint, as indicated by a 2.4‐fold higher plasma blood urea nitrogen (BUN) (Fig. 1 *A*) and serum creatinine (Fig. 1 *B*) levels in CKD compared with normal rats with no effect of treatment. Plasma phosphorus levels were higher in CKD animals compared with normal animals (Fig. 1 *C*), but plasma calcium levels were not different between animal groups (Fig. 1 *D*). The plasma levels of PTH (Fig. 2 *A*) were significantly higher than normal in all CKD groups. As expected, CKD rats treated with KP‐2326 had lower PTH levels compared with non‐treated CKD animals (1.9‐fold decrease in CKD/KP compared with CKD). In contrast, whereas PTH levels trended lower in combination‐treated animals, there was not a significant difference between the CKD and CKD/GKT + KP groups. CKD animals also demonstrated higher serum C‐terminal and intact FGF23 levels compared with normal animals (Fig. 2 *B, C*). Interestingly, serum cFGF23 was 1.7‐fold higher in CKD rats treated with GKT alone and when combined with KP compared with non‐treated CKD rats (Fig. 2 *B*). However, there was no difference in serum intact FGF23 levels between CKD‐treated and non‐treated animals (Fig. 2 *C*). This finding suggests that GKT treatment did not affect serum intact FGF23 levels but may facilitate FGF23 cleavage, leading to elevated serum cFGF23 in CKD rats. There was also higher oxidative stress as measured by 8‐OHdG, a marker of DNA oxidation, in CKD rats compared with normal animals with no treatment effect (Fig. 2 *D*).

### Effects of GKT and KP on the cardiovascular system

**Fig. 3:**
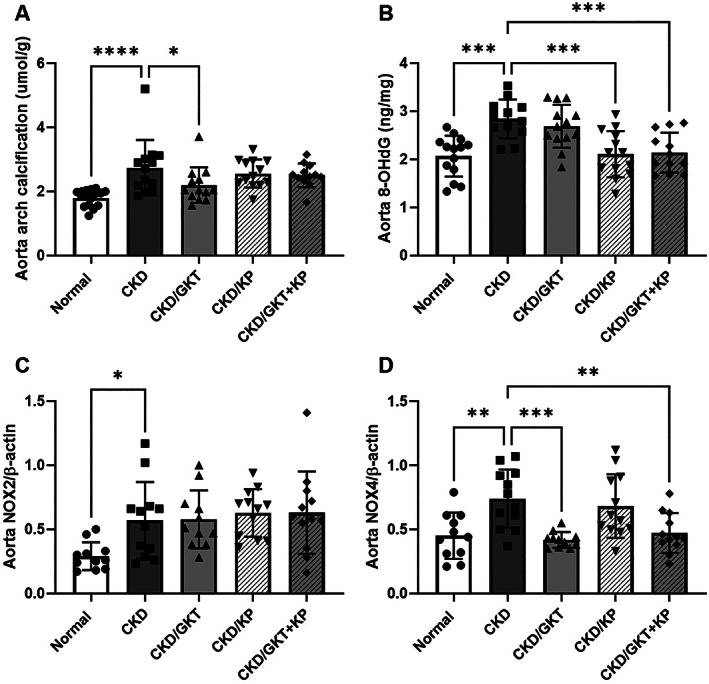
The effects of GKT and KP treatment on aortic calcification and oxidative stress. Aortic calcification was significantly elevated in CKD animals but was decreased by GKT treatment alone (*A*). 8‐OHdG levels in the aorta were increased in CKD and decreased in KP‐ and combination‐treated animals (*B*). Aortic NOX2 and NOX4 expression were increased in CKD, whereas GKT treatment significantly reduced NOX4 expression (*C, D*). Data are shown as mean ± SD and analyzed by one‐way ANOVA. If *p* < 0.05, Dunnett's multiple comparison test was performed for each group versus CKD: *p < 0.05, **p < 0.01, ***p < 0.001, ****p < 0.0001. NOX = nicotinamide adenine dinucleotide phosphate (NADPH) oxidase.

There was a 1.5‐fold higher level of aortic arch calcification in CKD animals compared with normal animals (Fig. 3 *A*). Treatment with GKT in CKD rats led to 1.2‐fold lower aortic arch calcification compared with non‐treated CKD (Fig. 3 *A*). However, this effect was negated in combination‐treated animals. In addition, the aortic tissue oxidative stress marker, 8‐OHdG, was 1.4‐fold higher in CKD animals compared with normal animals (Fig. 3 *B*). Treatment with KP alone and in combination with GKT, but not GKT alone, led to lower 8‐OHdG levels in the aorta compared with non‐treated CKD rats (Fig. 3 *B*). To further assess the effect of treatments on oxidative stress, we assessed the mRNA for superoxide dismutase 1 and 2, but found no effect by either treatment (Supplemental Fig. S1). However, aortic expression of NOX2 and NOX4 were higher in CKD animals compared with normal animals (Fig. 3 *C, D*), and treatment with GKT alone or in combination with KP in CKD rats led to lower NOX4 expression in the aorta but did not alter NOX2 expression compared with non‐treated CKD rats, demonstrating the efficacy and specificity of the inhibitor (Fig. 3 *D*). In the heart, there was higher calcification and LVMI in CKD animals compared with normal animals but no effect of treatments (Supplemental Table S1).

### Effects of GKT and KP on bone

**Fig. 4:**
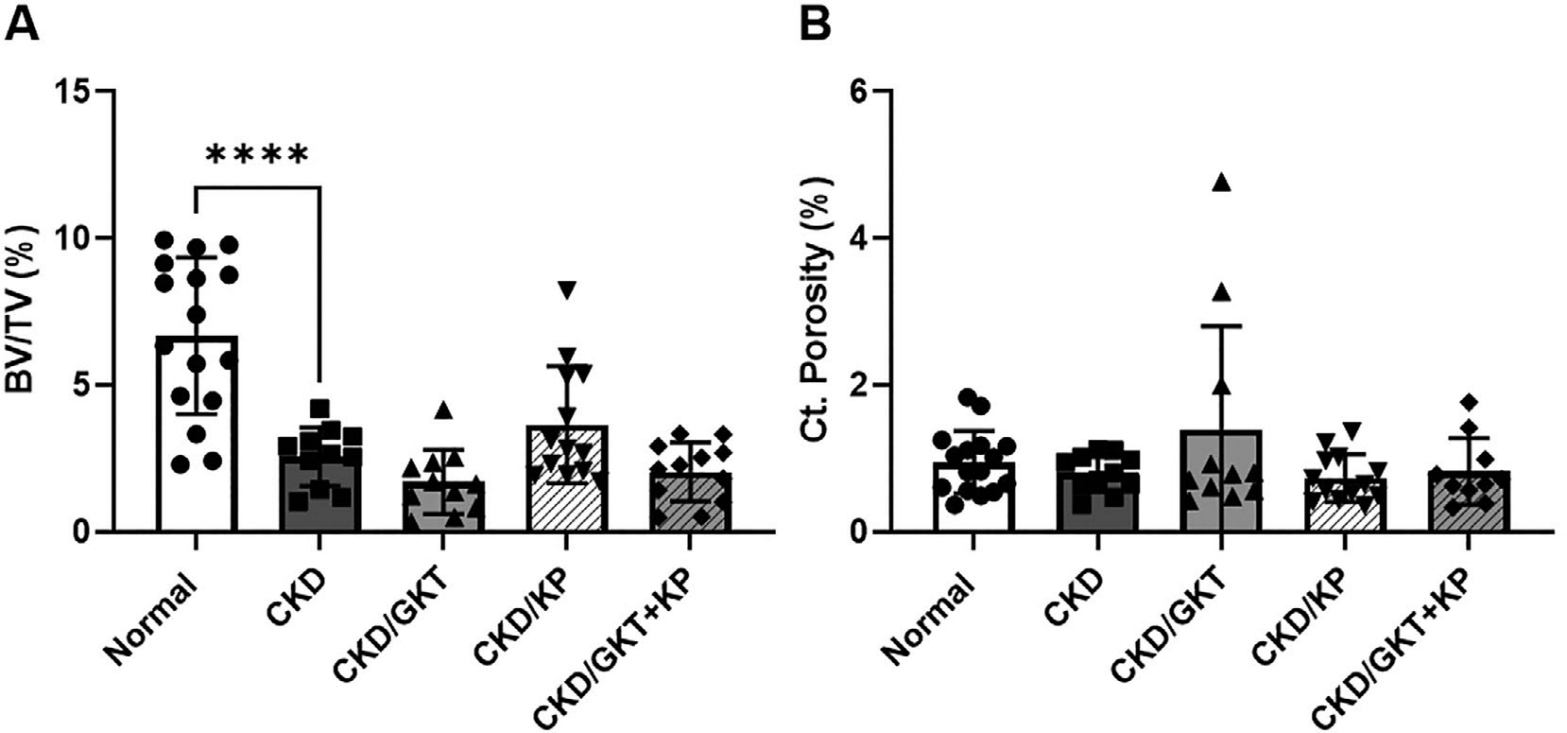
The effects of GKT and KP treatment on trabecular and cortical bone. Bone volume fraction of trabecular bone was significantly decreased in CKD animals compared with normal animals and was not altered by either treatment (*A*). Cortical porosity was not altered in CKD rats or by any treatments (*B*). Data are shown as mean ± SD and analyzed by one‐way ANOVA. If *p* < 0.05, Dunnett's multiple comparison test was performed for each group versus CKD: *p < 0.05, ****p < 0.0001. BV/TV = bone volume/total volume; Ct.Porosity = cortical porosity.

Micro‐CT assessment of the proximal tibia demonstrated lower trabecular bone volume fraction and trabecular number in CKD animals compared with normal animals (Fig. 4 *A*, Supplemental Table S2). The average trabecular thickness was unchanged by CKD and treatments, whereas the average separation between trabeculae was higher in CKD rats compared with normal rats (Supplemental Table S2). There was no effect of KP on trabecular bone geometry, whereas GKT further increased trabecular separation (Fig. 4 *A*, Supplemental Table S2). Analysis of cortical bone at proximal 1/3 of the tibia showed no cortical porosity (Fig. 4 *B*) in CKD rats, likely due to the earlier study endpoint and therefore less severe CKD compared with our previous studies.^(^ ^31^ ^)^ Furthermore, there were no changes in cortical bone area or cortical thickness in CKD animals compared with normal (Supplemental Table S2). Analysis of force‐displacement and stress‐strain mechanical data revealed a lower ultimate force but no differences in the tissue‐level properties in CKD animals compared with normal (Table 1). KP and/or GKT treatment did not alter any structural or tissue‐level mechanical properties compared with CKD animals.

**Table 1:** Mechanical properties of the femur

	Ultimate force (N)	Total displacement (mm)	Stiffness (N/mm)	Total work (mJ)	Ultimate stress (MPa)	Total strain (mε)	Modulus (GPa)	Toughness (MPa)
NL	302 ± 35**	775 ± 95	529 ± 98	140 ± 30	167 ± 19	42.7 ± 5.98	5.41 ± 1.31	4.27 ± 0.86
CKD	265 ± 23	772 ± 108	465 ± 66	125 ± 28	160 ± 21	41.1 ± 4.80	5.24 ± 0.94	4.04 ± 1.08
CKD/GKT	255 ± 33	808 ± 94	409 ± 81	124 ± 20	155 ± 25	43.8 ± 6.13	4.58 ± 1.01	4.02 ± 0.68
CKD/KP	273 ± 19	770 ± 82	502 ± 81	130 ± 17	168 ± 12	40.5 ± 5.73	5.96 ± 1.38	4.19 ± 0.53
CKD/GKT + KP	253 ± 25	769 ± 108	400 ± 75	114 ± 21	153 ± 17	40.5 ± 5.81	4.58 ± 0.82	3.62 ± 0.65

NL = normal; CKD = chronic kidney disease; GKT = GKT‐137831; KP = KP‐2326. Data are shown as mean ± SD and analyzed by one‐way ANOVA. Dunnett's multiple comparison test was performed for each group versus CKD.

** *p* < 0.01.

## Discussion

The CKD rat undergoes progressive kidney disease with a phenotype resembling human CKD‐MBD, including altered biochemistries and cardiovascular calcification with increased bone fragility. Our previous in vivo studies have demonstrated that freshly isolated VSMCs from our CKD rats have increased intracellular calcium,^(^ ^34^ ^)^ indicative of a synthetic phenotype necessary for differentiation to an osteochondritic phenotype for calcification.^(^ ^35^ ^)^ The elevation in intracellular calcium is likely due to the elevated PTH observed in our animals. Furthermore, we have shown that matrix vesicles isolated from normal rats are endocytosed by VSMCs from CKD rats and increase intracellular calcium and oxidative stress, and that calcification induced by the matrix vesicles can be inhibited by the NOX1/4 inhibitor GKT in vitro.^(^ ^22^ ^)^ Calcimimetics, allosteric activators of the calcium‐sensing receptor, lower PTH and can prevent arterial calcification in multiple animal models.^(^ ^14^, ^15^ ^)^ Therefore, we hypothesized that the combination of inhibition of NOX1/4 with GKT and reduction of PTH and intracellular calcium with the calcimimetic KP would have additive effects to reduce arterial calcification.

We confirmed our in vitro findings of reduced vascular calcification with GKT in vivo in the present study, demonstrating both a reduction in NOX4 expression and aorta calcification with GKT. We also saw the expected reduction of PTH and cFGF23 with calcimimetic treatment.^(^ ^36^ ^)^ However, the combination unexpectedly negated the GKT inhibition of calcification despite persistent suppression of NOX4, and negated the calcimimetic inhibition of PTH. Furthermore, GKT alone did not suppress 8‐OHdG, a marker of DNA oxidative stress, in the aorta or serum but KP alone and the combination reduced 8‐OHdG in the aorta without changing NOX4. The results suggest that in vivo, there is an interaction of GKT and KP. We hypothesize that the binding of KP to the calcium‐sensing receptor, which is known to increase intracellular calcium to prevent release of PTH,^(^ ^37^ ^)^ may have interfered with the efficacy of NOX4 inhibition. Prolonged increases in intracellular calcium can increase oxidative stress,^(38^ ^)^ and increased oxidative stress can increase intracellular calcium in VSMCs,^(39^ ^)^ creating a vicious cycle. Alternatively, additional NOX species may counteract the suppression of NOX4 when there is a change in intracellular calcium, as NOX1, NOX2, and NOX4 are differentially regulated.^(^ ^40^ ^)^ These results were surprising and suggest a more complicated cell signaling mechanism that would require acute measures of intracellular calcium and oxidative stress to confirm. However, the results are counter to our hypothesis of an additive effect.

Osteocytes secrete FGF23, which increases renal phosphorus excretion and suppresses 1,25(OH)_2_ vitamin D synthesis. It is now known that in CKD, there is altered cleavage of FGF23 in response to anemia, erythropoietin (EPO), and inflammation.^(^ ^41^ ^)^ Throughout the course of CKD, cFGF23 levels typically decrease, resulting in a large pool of active, intact FGF23.^(^ ^42^ ^)^ In this study, we observed increased cFGF23 in the serum of all CKD animals, increasing further with GKT with or without KP, but no effect of KP alone. However, intact levels of FGF23 were not affected by any treatment, which may indicate increased secretion without increased cleavage of FGF23 with NOX1/4 inhibition by GKT. This suggests that inhibition of NOX1/4 with GKT inhibits the normal CKD‐induced FGF23 cleavage. Hypoxia‐inducible factor‐α (HIFα) is upregulated in anemia and increases EPO production in the kidney but also increases NOX4 in pulmonary artery smooth muscle cells.^(^ ^43^ ^)^ Thus, it is plausible that GKT may interfere with HIFα effects on FGF23 cleavage. Unfortunately, we did not measure iron or EPO in this study; however, our animals are known to be anemic and iron deficient at 28 weeks of age (unpublished observation). More work is needed to understand this relationship.

Although previous studies from our group have demonstrated increased cortical porosity and cortical thinning with decreased trabecular bone volume fraction in 35‐week‐old CKD rats (stage 5 CKD or end stage), this study only observed trabecular bone alterations in 28‐week‐old animals.^(^ ^30^, ^44^ ^)^ We purposely studied our animals at an earlier time point as we hypothesized our interventions would prevent vascular calcification. This indicates that cortical bone changes may occur later in CKD, which is consistent with studies correlating decreased trabecular bone density with the progression of vascular calcification before cortical bone loss in early‐stage CKD patients.^(^ ^45^ ^)^ We also note that while PTH was elevated in CKD animals, these values are lower than what we have previously reported in CKD rats at 35 weeks of age.^(^ ^30^, ^31^ ^)^ PTH levels are correlated with cortical bone porosity in humans with CKD^(^ ^46^ ^)^ and in our animals.^(^ ^47^ ^)^ However, PTH suppression with KP, a preclinical analog of the peptide calcimimetic drug etelcalcetide, did not alter trabecular or cortical bone geometry (Fig. 4, Supplemental Table S2). However, it should be noted that while PTH was suppressed compared with untreated CKD animals, the average PTH level in KP‐treated animals was higher than that in normal animals. Therefore, if PTH impacts the trabecular bone compartment in CKD, further PTH suppression may be necessary to see these changes.

Although CKD negatively impacted trabecular bone, the only altered structural mechanical property in the femur was reduced ultimate force in CKD animals (Table 1). Trabecular bone was not significantly altered by KP or GKT treatment, and there were no improvements in mechanical properties in treated animals compared with untreated CKD animals. Further, the lack of differences in the transformed stress‐strain data indicates that the tissue‐level properties, including ultimate stress and modulus, were not altered in CKD animals. One limitation here is that these are approximated, not true tissue‐level measures of bone mechanics. Because of the progressive nature of CKD in Cy/+ rats, bone quality may be heterogeneous as bone is formed and remodeled across stages of CKD and while PTH levels are changing. Therefore, tissue‐level mechanical properties may be altered in subregions of the bone that contribute to the overall ultimate force but are not detected via stress‐strain transformation. Therefore, further studies are needed to measure the true tissue‐level properties of bone, including composition and material properties, while controlling for tissue age in CKD.

CKD animals treated with the NOX1/4 inhibitor GKT and the calcimimetic drug KP demonstrated decreased calcification, 8‐OHdG, and NOX4 expression in the vasculature, decreased PTH, and increased cFGF23. KP was responsible for lowering PTH and aortic 8‐OHdG, whereas GKT decreased NOX4 and calcification while increasing cFGF23. Counter to our hypothesis, these effects appear to be independent of one another in CKD animals, as there were no additive effects of the two treatments and, in fact, negated effects with single agent on vascular calcification and FGF23 levels. In the bone, CKD animals had lower trabecular volume that was not improved with calcimimetics. However, there were no changes in the cortical bone, the most common bone compartment affected by hyperparathyroidism, likely reflecting the early time point of intervention/assessment. Inhibition of NOX1/4 decreased arterial calcification in vivo, but its efficacy was inhibited, not augmented, with simultaneous lowering of PTH with a calcimimetic. Taken together, these results suggest that NOX1/4 clearly plays a role in arterial calcification in vivo, confirming our in vitro results. However, the addition of a calcimimetic reduced the efficacy, likely due to an interaction in cell signaling pathways, but the mechanism remains to be defined.

## Disclosures

SMM provides scientific consultation for Amgen, Sanifit, and Ardelyx and receives funding from Chugai and Keryx. MRA receives funding from Amgen. All other authors state that they have no conflicts of interest.

### Peer review

The peer review history for this article is available at https://publons.com/publon/10.1002/jbm4.10600.

## Supporting information


**Supplemental Fig. S1.** The effects of GKT and KP treatment on the mRNA expression of antioxidants superoxide dismutase‐1 (SOD‐1; left panel) and superoxide dismutase‐2 (SOD‐2; right panel). Data are shown as mean ± SD and analyzed by one‐way ANOVA.

**Supplemental Table S1.** Left Ventricular Calcification and Mass
**Supplemental Table S2.** Trabecular and Cortical Microarchitecture of the Tibia
