# Supplementary material for: Non‐Additive Effects of Combined NOX1/4 Inhibition and Calcimimetic Treatment on a Rat Model of Chronic Kidney Disease‐Mineral and Bone Disorder (CKD‐MBD)
Source: JBMR Plus. 2022 Feb 11;6(3):e10600. doi: 10.1002/jbm4.10600 (PMC8914155; doi:10.1002/jbm4.10600)
Supplement: Supplementary file 1 — Supplemental Fig. S1. The effects of GKT and KP treatment on the mRNA expression of antioxidants superoxide dismutase‐1 (SOD‐1; left panel) and superoxide dismutase‐2 (SOD‐2; right panel). Data are shown as mean ± SD and analyzed by one‐way ANOVA. [file JBM4-6-e10600-s002.zip › Supplemental information Figure 1.docx]

**Supplemental Figure 1**: The effects of GKT and KP treatment on the mRNA expression of anti-oxidants superoxide dismutase-1 (SOD-1 left) and superoxide dismutase-2 (SOD-2 right panel). Data are shown as mean ± SD and analyzed by One-way ANOVA.
