# Supplementary material for: Non‐Additive Effects of Combined NOX1/4 Inhibition and Calcimimetic Treatment on a Rat Model of Chronic Kidney Disease‐Mineral and Bone Disorder (CKD‐MBD)
Source: JBMR Plus. 2022 Feb 11;6(3):e10600. doi: 10.1002/jbm4.10600 (PMC8914155; doi:10.1002/jbm4.10600)
Supplement: Supplementary file 2 — Supplemental Table S1. Left Ventricular Calcification and Mass Supplemental Table S2. Trabecular and Cortical Microarchitecture of the Tibia [file JBM4-6-e10600-s001.docx]

| **Supplementary Table 1.** Left ventricular calcification and mass | | |
| --- | --- | --- |
|  | Heart Calcification (μmol/g) | LVMI |
| NL | 0.45 ± 0.08^***^ | 2.79 ± 0.17^*^ |
| CKD | 0.59 ±0.16 | 2.99 ± 0.16 |
| CKD/GKT | 0.60 ± 0.07 | 3.10 ± 0.32 |
| CKD/KP | 0.52 ± 0.06 | 2.97 ± 0.14 |
| CKD/GKT+KP | 0.58 ± 0.09 | 3.01 ± 0.18 |
| Data are shown as mean ± SD and analyzed by One-way ANOVA. Dunnett’s multiple comparison test was performed for each group versus CKD: ^*^*p* < 0.05; ^***^*p* < 0.001. LVMI = left ventricular mass index. | | |

| **Supplementary Table 2.** Trabecular and Cortical Microarchitecture of the Tibia | | | | | | | |
| --- | --- | --- | --- | --- | --- | --- | --- |
|  | BV/TV (%) | Tb.N (#/mm) | Tb.Th (mm) | Tb.Sp (mm) | Ct.Porosity (%) | Ct.Th (mm) | Bone Area (mm^2^) |
| NL | 6.68 ±2.7^****^ | 0.84 ± 0.3^****^ | 0.08 ± 0.01 | 0.77 ± 0.09^****^ | 0.95. ± 0.42 | 0.51 ± 0.02 | 7.01 ± 0.46 |
| CKD | 2.56 ± 1.0 | 0.30 ± 0.1 | 0.09 ± 0.01 | 0.54 ± 0.10 | 0.80 ± 0.25 | 0.50 ± 0.02 | 6.67 ± 0.24 |
| CKD/GKT | 1.71 ± 1.1 | 0.21 ± 0.1 | 0.08 ± 0.01 | 0.86 ± 0.07^*^ | 1.40 ± 1.41 | 0.47 ± 0.06 | 6.33 ± 0.33 |
| CKD/KP | 3.65 ± 2.0 | 0.40 ± 0.2 | 0.09 ± 0.01 | 0.74 ± 0.08 | 0.74 ± 0.32 | 0.52 ± 0.03 | 6.83 ± 0.38 |
| CKD/GKT+KP | 2.04 ± 1.0 | 0.24 ± 0.1 | 0.08 ± 0.01 | 0.84 ± 0.10 | 0.82 ± 0.45 | 0.49 ± 0.04 | 6.58 ± 0.55 |
| Data are shown as mean ± SD and analyzed by One-way ANOVA. Dunnett’s multiple comparison test was performed for each group versus CKD: ^*^*p* < 0.05; ^****^*p* < 0.0001. BV/TV = bone volume/total volume; Tb.N = trabecular number; Tb.Th = trabecular thickness; Tb.Sp = trabecular separation; Ct.Porosity = cortical porosity; Ct.Th = cortical thickness. | | | | | | | |
